# New Insights into the Loss of Antioxidant Effectiveness of Phenolic Compounds in Vegetable Oils in the Presence of Phosphatidylcholine

**DOI:** 10.3390/antiox12111993

**Published:** 2023-11-11

**Authors:** Joaquín Velasco, María-Jesús Gil, Yun-Qi Wen, Aída García-González, María-Victoria Ruiz-Méndez

**Affiliations:** 1 Instituto de la Grasa, Consejo Superior de Investigaciones Científicas (CSIC), Ctra. de Utrera, km 1, 41013 Sevilla, Spain; mjgil@ig.csic.es (M.-J.G.); aida.garcia@csic.es (A.G.-G.); mvruiz@ig.csic.es (M.-V.R.-M.); 2 College of Food Science and Technology, Ocean University of China, No. 5 Yu Shan Road, Qingdao 266003, China; wenyq0715@163.com

**Keywords:** reverse micelles, lipid oxidation, antioxidant, phosphatidylcholine, α-tocopherol, Trolox, sunflower oil, Rancimat

## Abstract

It has been proposed that lipid oxidation reactions in edible oils primarily occur in reverse micelles (RM) of amphiphilic components. While the prooxidative effect of RM has been demonstrated, the mechanism involved is not fully understood. Both reductions and enhancements in the antioxidant efficacy (AE) of α-tocopherol and Trolox have been observed in different studies when phosphatidylcholine (PC) was added and PC RM were formed. However, most of these investigations employed lipid systems consisting of stripped vegetable oil diluted in saturated medium-chain triacylglycerols (MCT) and utilized antioxidant concentrations well below those found in edible oils. These two specific factors were investigated in the present study. The effect of RM of purified egg yolk PC on the AE of 1.16 mmol kg^−1^ α-tocopherol or Trolox in stripped sunflower oil (SSO) was studied by the Rancimat (100 °C) and oven (50 °C) tests. Increasing PC concentrations (50–1000 ppm) had no significant impact on α-tocopherol, but substantial reductions in AE were observed for Trolox. This phenomenon may be attributed to the partitioning of Trolox into the pre-existing PC micelles, suggesting that primary oxidation reactions occurred in the continuous lipid phase. In addition, the effectiveness of both antioxidants decreased significantly in the presence of PC when a low antioxidant concentration (0.06 mmol kg^−1^) was assayed in SSO:MCT (1:3, *w*/*w*).

## 1. Introduction

Edible oils comprise triacylglycerols in around 98 wt% and a variety of minor components, included sterols, hydrocarbons, free fatty acids, diacylglycerols, monoacylglycerols, tocopherols, and phospholipids. Many of these are amphiphilic and they can associate with water trace, whose contents can oscillate between 200 and 2000 mg kg^−1^ in refined oils [1]. In turn, they self-aggregate to form reverse micelles, in which the surface-active molecules orientate with their polar heads towards the aqueous core of the micelles and the nonpolar tails towards the apolar continuous phase [1]. These nanoscale colloidal units change the physical and chemical properties of oils and have an impact on lipid oxidation [2,3]. Through complex reactions, unsaturated fatty acyl chains react with oxygen and give rise to a number of compounds that impart off-flavors and make the oil unacceptable to consumers [4].

It has been demonstrated that reverse micelles in oils play a catalytic role in lipid oxidation [2]. It was suggested that hydroperoxides accumulate at the interfacial region of these colloids due to their interfacial activity and decompose into free radicals by the action of transition metals concentrated at the interface. However, the prooxidative mechanism involved is not fully understood. Different results have been obtained in a number of reports. Dioleylphosphatidylcholine (DOPC) association colloids promoted both primary and secondary lipid oxidation in purified or stripped soybean oil (SSbO), and mixtures of SSbO or stripped corn oil (SCO) with saturated medium-chain triacylglycerols (MCT) [5,6,7,8,9]. In addition, a mixture of surface-active components commonly present in oils, such as DOPC, dioleylphosphatidylethanolamine (DOPE), diolein, oleic acid, and stigmasterol, also formed reverse micelles in blends of SCO and MCT and promoted lipid oxidation at concentrations above the critical micelle concentration (CMC) [10]. In contrast, no effects were found for similar mixed reverse micelles in stripped high oleic or high linoleic safflower oils [11].

It has been stated that the greater antioxidative efficacy of polar antioxidants in bulk oils, compared to lipophilic antioxidants, may be attributed to their greater affinity for the water–oil interfaces created by reverse micelles of oil minor components, where oxidative reactions appear to predominate [1,2].

A comprehensive understanding of the physicochemical mechanisms of antioxidants in bulk oils is of utmost importance to enhance their effectiveness and achieve better control of lipid oxidation. Interactions between antioxidants and association colloids, and particularly their placement in the micellar structure, can significantly impact their antioxidant efficacy [2]. Koga and Terao postulated that phospholipids enhanced the accessibility of α-tocopherol to the free radicals generated by a water-soluble radical initiator in a bulk oil model system, improving its antioxidant activity [12]. They affirmed that α-tocopherol was able to scavenge the chain-initiating free radicals generated in an aqueous microenvironment and the chain-propagating peroxyl radicals in the continuous lipid phase.

Studies on the effect of reverse micelles on the efficacy of antioxidants, such as α-tocopherol and its synthetic polar homologue Trolox, have shown varying results depending on the antioxidant concentration. DOPC reverse micelles improved the activity of low α-tocopherol or Trolox concentrations (10 µM) in a blend of SSbO and MCT (1:3, *w*/*w*) [6], but decreased the activity of high antioxidant concentrations (100 µM) [6,13,14]. A synthetic phospholipid unable to form reverse micelles, such as 1,2-dibutyryl-*sn*-glycero-3-phosphocholine (DC_4_PC), also decreased the antioxidant activity of both α-tocopherol or Trolox in SSbO:MCT (1:3, *w*/*w*) at low (10 µM) and high (100 µM) concentrations [6].

For convenience, a common practice in these studies is the use of a stripped vegetable oil diluted in MCT oil as a lipid substrate (e.g., [6,13,14]). This approach allows one to prepare a large volume of samples in a short time because MCT oil does not require any tedious purification step to remove naturally occurring antioxidants and other amphiphilic minor oil components. In addition to the purification advantage, the use of MCT allows testing antioxidants at very low concentrations. The degree of unsaturation is considerably reduced with the addition of MCT, which comprises saturated medium-chain fatty acids, resulting in increased oxidative stability. To shorten the time scale of oxidation experiments, antioxidants are used at very low concentrations. This model system may not accurately represent common vegetable oils, which typically contain much higher concentrations of natural antioxidants, such as tocopherols. For example, the concentration of α-tocopherol in refined sunflower oil ranges between 400 and 900 mg kg^−1^ [15] or between 854 µM and 1922 µM, considering that the oil density is 0.92 kg L^−1^ [15]. More importantly, the interactions between antioxidants and reverse micelles may be overstated due to the extremely high differences in concentrations between reverse micelle components and antioxidants.

The main goal of the present work was to study in more detail the effect of reverse micelles on the antioxidant efficacy of phenolic antioxidants in bulk oil using a model system which was closer to refined vegetable oils. Neat stripped sunflower oil (SSO) was chosen as lipid substrate, and purified egg yolk phosphatidylcholine (PC) was selected as the surfactant. PC was chosen because it plays a major role as a surface-active component in the formation of reverse micelles in oils [10]. The effect of increasing concentrations of PC on the effectiveness of α-tocopherol at concentrations normally found in sunflower oil was studied. For comparative purposes, Trolox, the polar homologue of α-tocopherol, was also investigated. The Rancimat test at 100 °C was the method applied to evaluate the effectiveness of antioxidants and results were validated by oxidation experiments in an oven at 50–60 °C (oven test). For comparative purposes, the effect of PC on the effectiveness of the antioxidants was also evaluated at conditions that were similar to those in reported studies [6,12,13,14]. In this regard, a very low concentration of the antioxidants (0.06 mmol kg^−1^) was assayed in SSO:MCT (1:3, *w*/*w*).

## 2. Materials and Methods

### 2.1. Materials

High linoleic sunflower oil was acquired from a local supermarket. Pure C8 medium chain triacylglycerol (MCT) oil (100% coconut sourced) was purchased from Ketosource B.V. (Amsterdam, The Netherlands). (±)-α-Tocopherol (>97%), (±)-6-hydroxy-2,5,7,8-tetramethylchormane-2-carboxylic acid (Trolox) (97%), gallic acid, propyl gallate (≥98%), dodecyl gallate, L-α-phosphatidylcholine from egg yolk (~60%), 7,7,8,8-tetracyanoquinodimethane (TCNQ) (98%), and hydranal^®^ Coulomat oil were acquired from Sigma-Aldrich (St. Louis, MO, USA). Aluminum oxide 90 active neutral was purchased from Merck KGaA (Daemstadt, Germany). n-Hexane (95%) (HPLC grade), chloroform stabilized with 0.5–0.8% ethanol (HPLC grade), methanol for UV, IR, HPLC, ACS, n-heptane (HPLC grade), and anhydrous methanol (max. 0.005% water) were obtained from Panreac Química S.L.U. (Barcelona, Spain), diethyl ether stabilized with 1% (*v*/*v*) ethanol (Super purity solvent, HPLC grade) was from Romil, LTD (Cambridge, UK), 2-propanol (gradient grade for HPLC) from Merck KGaA (Daemstadt, Germany), and dichloromethane (≥99.8%, HPLC grade) from Honeywell GmbH (Seelze, Germany). Except for the L-α-phosphatidylcholine, the chemicals and solvents were used as received.

### 2.2. Oil Stripping Procedure

In order to remove the minor components naturally present in the oil, the sunflower oil was purified by adsorption chromatography using a glass column packed with aluminum oxide according to Fuster et al. [16]. Briefly, the oil was dissolved in an equal volume of n-hexane and passed through the column containing alumina activated at 200 °C during 20 h and conditioned with a volume of n-hexane equal to its mass. Nitrogen was bubbled through the eluate over the process. The solvent was removed in a Büchi R-210 rotary evaporator (Büchi Ibérica, Barcelona, Spain) at 35 °C and finally by bubbling the oil with nitrogen at room temperature. The oil was protected from light with aluminum foil during all the procedure and it was preserved under nitrogen at −20 °C until analyses. According to Fuster et al. [16], the purification method gives rise to an oil containing undetectable amounts of tocopherols, peroxides, and iron and copper levels below 0.01 and 0.001 ppm, respectively. The absence of tocopherols [17], hydroperoxides [18], free fatty acids [19], and mono- and di-acylglycerols [19] were verified in the present study by HPLC.

### 2.3. Phosphatidylcholine Purification

L-α-phosphatidylcholine (PC) from egg yolk was selected owing to its high level of PC (~60%) and low price. It was purified by adsorption chromatography using a glass column packed with alumina 90 according to Singleton et al. [20]. The alumina was not activated and used as received. The elution solvent was chloroform:methanol 9:1 (*v*/*v*). This was also used for packing and conditioning the alumina. A fraction collector was used and the progress of the fractionation was followed by thin layer chromatography (TLC), employing 50% sulfuric acid to reveal the spots. The solvent was evaporated in the rotary evaporator at 35 °C and finally with a nitrogen stream at room temperature until dryness. The PC so obtained was chromatographically pure. This was preserved under nitrogen at −20 °C until analysis. The fatty acid composition of PC was determined by standard IUPAC methods 2.301 and 2.302, applying the special preparation of the methyl esters of fatty acids with potassium hydroxide in methanol at room temperature [21]. The fatty acid composition of PC did not change with the purification and their main fatty acids were 0.14% C14:0, 30.49% C16:0, 0.26% C16:1 n-9, 1.26% C16:1 n-7, 0.16% C17:0, 12.29% C18:0, 28.70% C18:1, 17.10% C18:2, 0.32% C18:3, 0.27% C20:3 n-6, 4.36% C20:4 n-6, 0.27% C22:4 n-6, 1.51% C22:5 n-6, and 1.26% C22:6 n-3.

### 2.4. Sample Preparation

Stripped sunflower oil (SSO) or a blend of SSO and MCT oil (SSO-MCT) in proportions 1:1 (*w*/*w*) or 1:3 (*w*/*w*) were the lipid substrates used in this study. The MCT oil was not purified, but it was dried at 80 °C and 100 mbar in a Büchi R-210 rotary evaporator (Büchi Ibérica, Barcelona, Spain) due to its elevated moisture content (ca. 1000 mg kg^−1^). The moisture content was reduced to 50 mg kg^−1^, which was virtually the same as that measured for the SSO.

The antioxidants were added to the oils using solutions in organic solvents. The antioxidant concentrations of the solutions were adjusted to add only 1 mL solution to an amount of oil that ranged between 40 and 240 g. α-Tocopherol was dissolved in n-heptane, whereas the rest of the antioxidants tested were dissolved in anhydrous methanol. In order to avoid unnecessary additional variables, 1 mL of anhydrous methanol was added to the oils stabilized with α-tocopherol and 1 mL of n-heptane to the rest of the samples when antioxidants were compared. After the antioxidant addition, the oils were stirred for 10 min under nitrogen and the solvent was removed at 35 °C and vacuum conditions (100 mbar) in the rotary evaporator.

The PC was dissolved in the oils using a dichloromethane solution of the phospholipid (40 mg mL^−1^). After addition of the PC, vigorous stirring was applied under nitrogen for 10 min. The solvent was removed at 35 °C and vacuum conditions (100 mbar) in the rotary evaporator. From concentrations above 1000 mg kg^−1^, the PC was not completely soluble in the SSO and the samples presented noticeable turbidity and solid particles in suspension. PC concentrations above the solubility limit were only assayed in the Rancimat test.

In the last step, the moisture content in the oils was adjusted to 200 ppm mg kg^−1^. Deionized Milli-Q water was added and vigorous stirring was applied under nitrogen for 5 h. The moisture content was measured using a C-20 Coulometric Karl-Fischer Titrator from Mettler Toledo S.A.E. (Barcelona, Spain). Hydranal^®^—Coulomat oil from Sigma-Aldrich (St. Louis, MO, USA) was the reactive employed.

### 2.5. Determination of the Critical Micelle Concentration (CMC)

The CMC was measured by applying the spectrophotometric assay proposed by Kanamoto, Wada, Miyajima, and Kito [22]. It is based on charge transfer interactions between TCNQ and phospholipids. TCNQ has a low solubility in oils, but this increases in the presence of reverse micelles. The absorbance of TCNQ at 480 nm increases drastically when phospholipids reverse micelles form. The CMC was taken as the break in the curve between the absorbance and the logarithm of phospholipid concentration.

### 2.6. Rancimat Test

The oxidative stability index (OSI) was determined at 100 °C or 70 °C (SSO with no antioxidants) in the Rancimat test using a 679 Rancimat device from Metrohm (Herisau, Switzerland), according to AOCS Cd 12b-92 method [23]. An amount of 2.5 g oil and 20 L h^−1^ air were used.

### 2.7. Oven Test

The oil samples (15 g) were placed into 250-mL amber glass bottles and these were tightly closed with a lid and screw cap. The specific area of the oil samples was 1 cm^−1^. Each sample was oxidized in triplicate. The bottles were placed in an oven at 50 °C or 60 °C (±0.1) and sample aliquots were taken periodically. The oxidation was monitored by the direct analysis of triglycerides containing linoleate hydroperoxides or hydroperoxydienes by HPLC-DAD following the method proposed by Velasco et al. [18] as a measure of primary oxidation. The HPLC chromatograph, the chromatographic conditions, and hydroperoxydiene quantitation can be found in detail in [18]. In addition, triglyceride polymers were also analyzed by High-Performance Size-Exclusion Chromatography (HPSEC) as a measure of advanced oxidation, applying ISO 16931:2009 standard method [19]. A 1200 AT HPLC chromatograph from Agilent Technologies (Agilent Technologies, Palo Alto, CA, USA) equipped with two Agilent HPSEC columns containing respectively 100- and 500-Å particles of highly cross-linked styrene-divinylbenzene copolymers, connected in series, and a refractive index detector was used. The separation was performed at 35 °C. Tetrahydrofuran at 1 mL min^−1^ was the elution solvent. The HPSEC chromatograms were also employed to confirm the absence of di- and mono-acylglycerols, and free fatty acids in the SSO. In fact, oil compounds with lower molecular weight than triacylglycerols were not detected in the analysis.

### 2.8. Statistical Analyses

All experiments were run in triplicate. Results were expressed as means and standard deviations (*n* = 3). One-factor analysis of variance was applied. Levene’s test based on the mean value was used to verify that variances were equal across groups. For multiple mean comparisons, Duncan’s test was used. Comparisons between two samples were made by Student’s *t*-test. Significance was defined at *p* < 0.05. The analyses were performed using 29.0 IBM SPSS Statistics program (IBM Corp., Chicago, IL, USA).

## 3. Results

### 3.1. Effect of PC on the Antioxidant Effectiveness of α-Tocopherol and Trolox in SSO

α-Tocopherol is by far the major and most important chain-breaking antioxidant in refined sunflower seed oil, with a typical concentration ranging between 400 and 900 mg kg^−1^ [15]. During the refining process of vegetable oils, phospholipids are removed to levels below 10 mg kg^−1^ of phosphorus. This limit corresponds to 230 mg kg^−1^ of phospholipids when applying the conversion factor of 23.0 proposed by Carelli et al. [24] to transform phosphorus into phospholipid content in degummed sunflower oils. This estimate is coherent with the content of 254 mg kg^−1^ obtained when phosphorus is expressed as dioleylphosphatidylcholine, whose molecular weight is 786.1. Therefore, levels higher than 300 mg kg^−1^ of phospholipids are not expected in refined oils.

The antioxidant efficacy (AE) of α-tocopherol in stripped sunflower oil (SSO) containing varying concentrations of pure egg yolk phosphatidylcholine (PC) was evaluated. The concentration of the antioxidant tested was that normally found in refined sunflower oil (500 mg kg^−1^), whereas that of the phospholipid ranged between 50 to 10,000 mg kg^−1^, i.e., from levels that can be found in commercial oils to those much higher than expected. For comparative purposes, Trolox, the hydrophilic counterpart of α-tocopherol, was also tested at the same experimental conditions, using the same molar concentration of 1.16 mmol kg^−1^ as α-tocopherol.

**Figure 1:**
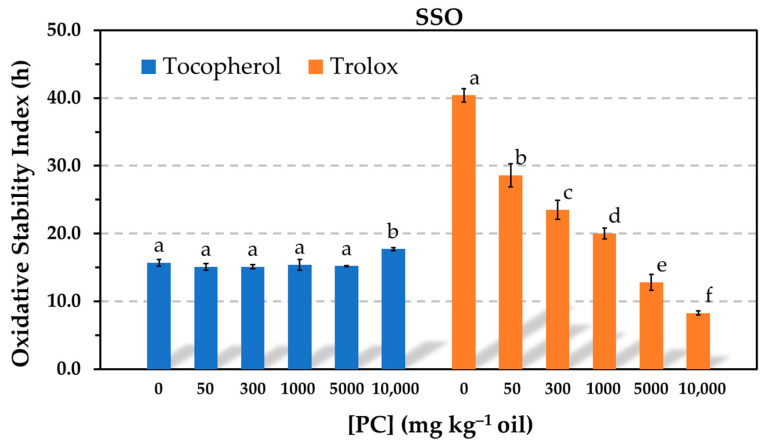
Influence of the phosphatidylcholine (PC) concentration on the oxidative stability index determined in the Rancimat test at 100 °C in stripped sunflower oil containing 1.16 mmol kg^−1^ α-tocopherol (500 mg kg^−1^) or Trolox (290 mg kg^−1^). Data represent means and standard deviations (*n* = 3). For a given antioxidant, different letters indicate significant differences according to Duncan’s test (*p* < 0.05).

The Rancimat test run at 100 °C was one of the methods selected in this study to evaluate the AE, because it allows one to make a number of experiments in a relatively short time. The results showed that the oxidative stability index (OSI) of the oil containing α-tocopherol did not significantly change in the presence of PC in a wide range of phospholipid concentrations (50–5000 mg kg^−1^) (Figure 1). A slight increase was only observed at the highest PC concentration tested, when a great amount of the phospholipid was not dissolved and the sample presented solid particles in suspension. The solubility limit of PC in the oil was found to be 1000 mg kg^−1^. Conversely, the OSI of the oil containing Trolox substantially decreased in the presence of PC in a concentration-dependent way (Figure 1). Nevertheless, Trolox was more effective than α-tocopherol when the PC was completely dissolved in the oil. The reductions in effectiveness observed for Trolox were consistent with most studies in this field, which reported the prooxidative activity of PC at levels above the critical micelle concentration (CMC) [3,5,6]. In contrast, the results obtained for α-tocopherol at a concentration normally found in commercial sunflower oil differ from those by other authors, who assayed the antioxidant at much lower concentrations [6,13,14].

**Figure 2:**
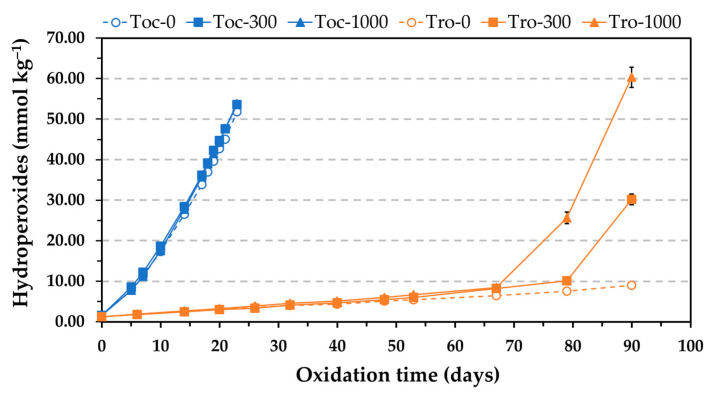
Influence of the phosphatidylcholine (PC) concentration on the formation of hydroperoxides during the oxidation at 50 °C of stripped sunflower oil containing 1.16 mmol kg^−1^ α-tocopherol (500 mg kg^−1^) or Trolox (290 mg kg^−1^). Toc and Tro in the sample codes represent α-tocopherol and Trolox, respectively, and the number after the hyphen indicates the PC concentration expressed in mg kg^−1^. Data represent means and standard deviations (*n* = 3). Some of the error bars are within data points.

The Schaal oven test was the other method used to evaluate the AE. This method is commonly used in studies addressed to investigate the role of reverse micelles of amphiphilic oil components in lipid oxidation. Oxidation experiments are normally run at 55 °C, and the levels of hydroperoxides and hexanal are measured to evaluate the extent of oxidation [6,13,14]. The Rancimat test applies higher temperatures to accelerate lipid oxidation, normally 100 °C for sunflower oils and other common vegetable oils [25]. In addition, the oil is oxidized by continuous forced aeration, which is achieved by bubbling air through it. To ensure non-limiting oxygen conditions, a considerable flow of air (15–20 L h^−1^) is applied, which makes the oil oxidize in a rough continuous movement. These particular dynamic conditions differ from the ones in the oven test, where the oil is oxidized at static conditions. Consequently, owing to the different experimental conditions applied in the Rancimat and oven tests, the AE of α-tocopherol and Trolox in the presence of PC was also evaluated at 50 °C by heating selected samples in an oven. The levels of hydroperoxides were measured to determine the oxidation extent. The results obtained at 50 °C were consistent with those by the Rancimat test. The levels of hydroperoxides in the sample containing α-tocopherol were not affected by the addition of PC at concentrations of 300 and 1000 mg kg^−1^, whereas hydroperoxides increased more rapidly with the addition of PC in the sample containing Trolox, showing a clear reduction in AE that was dependent on the PC concentration (Figure 2). Therefore, these results supported those obtained by the Rancimat test.

In order to evaluate whether the antioxidants had an impact on the PC reverse micelle formation at such a relatively high concentration (1.16 mmol kg^−1^), so far undocumented, the CMC was determined in SSO at the temperatures used in the Rancimat and oven tests. The TCNQ method applied presented a few issues. In the absence of antioxidants, the samples rapidly faded and no clear changes in color were noticed over a wide range of PC concentrations (0–1000 mg kg^−1^), neither at 50 °C nor at 100 °C. While a sharp rise in the absorbance was found in the presence of Trolox when the PC concentration was increased, the absorbance only increased moderately, and also unevenly, in the presence of α-tocopherol, which made it difficult to determine the CMC (Figure S1). The CMC did not show changes with temperature, and it was found to be 60–80 mg kg^−1^ PC when Trolox was tested. The absorbance increments also appeared to occur in the same range of PC concentrations in the presence of α-tocopherol. Even though the reactant was more soluble in the presence of Trolox when the PC micelles were formed, as higher absorbances were found compared to α-tocopherol (Figure S1), these results suggest that the type of antioxidant did not affect the CMC. Therefore, the CMC determined using the TCNQ method was unaffected by both the type of antioxidant and the temperature (50 °C compared to 100 °C).

The CMC found in this study is coherent with the result reported by Laguerre et al. [26] for dioleylphosphatidylcholine (DOPC) in stripped corn oil (SCO), which was found to be 65 µM at 55 °C, or 56 mg kg^−1^ considering that the density of SCO is 0.92 kg L^−1^. By small-angle X-ray scattering (SAXS) measurements, Chen et al. [6] found that the presence of α-tocopherol or Trolox, assayed at much lower concentrations (100 µM) than that of the present study (1.16 mmol kg^−1^ or 1067 µM considering that the density of SSO is 0.92 kg L^−1^), did not have any impact on the structure of DOPC reverse micelles in stripped soybean oil.

**Figure 3:**
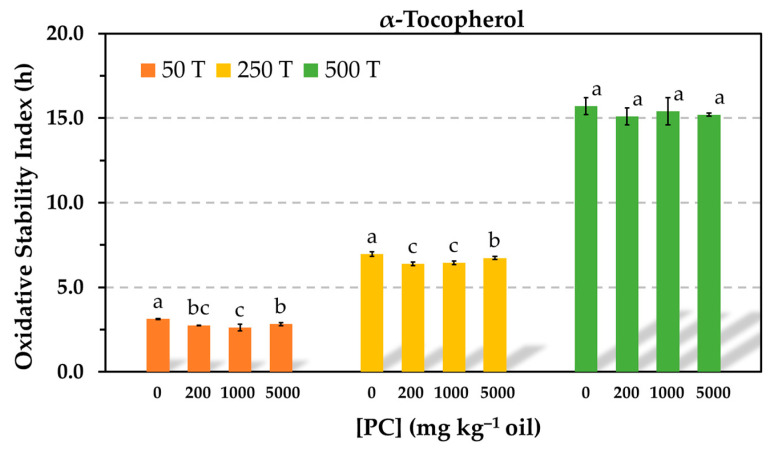
Influence of the phosphatidylcholine (PC) concentration on the oxidative stability index determined in the Rancimat test at 100 °C in stripped sunflower oil containing 50 (50 T), 250 (250 T), or 500 (500 T) mg kg^−1^ α-tocopherol. Data represent means and standard deviations (*n* = 3). For a given antioxidant concentration, different letters indicate significant differences according to Duncan’s test (*p* < 0.05).

Despite the limitations of the TCNQ method to measure accurately the CMC, which is based upon the intersection of two linear regression curves that depend on the number and selection of data points, the results of this study exhibited that the antioxidant activity of Trolox was significantly reduced even when the PC was tested at a concentration below the CMC and that the antioxidant activity of α-tocopherol was not affected even in the presence of PC reverse micelles. Compared to studies reported by other authors, the main difference of the present study is that the antioxidants were tested at much higher concentrations to study a model that is closer to a commercial oil. It is evident that interactions between the antioxidants and phospholipids depend on their relative concentrations. In order to study the impact of PC on the antioxidant activity of α-tocopherol in more detail, similar experiments were run in the Rancimat test using decreased α-tocopherol concentrations. When the antioxidant concentration was reduced ten-fold, the PC had an effect on the oil stability, but this was only marginal. Very slight but significant losses of stability were detected in the presence of PC. The same was true when the antioxidant concentration was halved (Figure 3).

### 3.2. Effect of PC on the Antioxidant Effectiveness of α-Tocopherol and Trolox at Reduced Concentration in a Blend of SSO and MCT (1:3, w/w)

**Figure 4:**
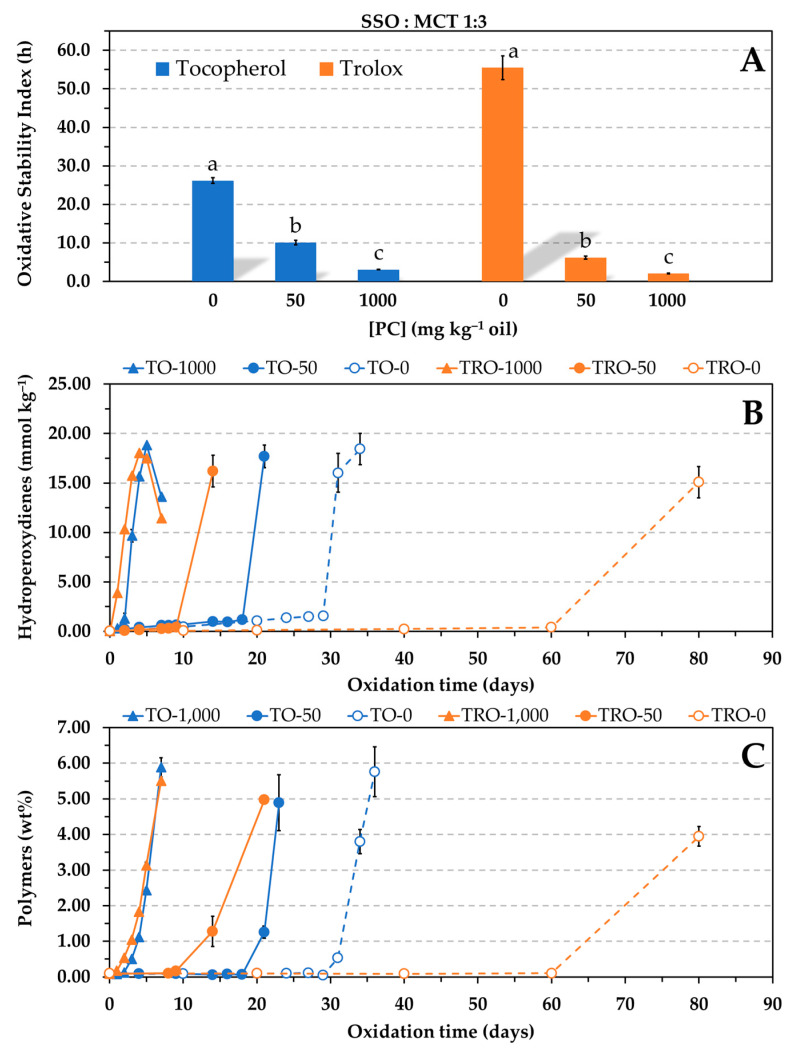
Influence of the phosphatidylcholine (PC) concentration on the antioxidant efficacy of α-tocopherol and Trolox at reduced concentration (0.06 mmol kg^−1^) in a blend of SSO and MCT (1:3, *w*/*w*). Oxidative stability index determined in the Rancimat test at 100 °C (**A**). Formation of hydroperoxides (**B**) and polymers (**C**) at 60 °C in an oven. Data represent means and standard deviations (*n* = 3). For a given antioxidant, different letters in (**A**) indicate significant differences according to Duncan’s test (*p* < 0.05). Some of the error bars in (**B**,**C**) are within data points.

Concentrations of α-tocopherol in SSO below 50 mg kg^−1^ were ruled out in the Rancimat test owing to the expected low OSI values. According to the AOCS official method [23], time periods less than 4 h in the Rancimat test result in a wider variation of the end point determination. To test a lower concentration of the antioxidant in a fast way and so confirm the loss of effectiveness of α-tocopherol in the presence of PC, the SSO was diluted in saturated medium-chain triacylglycerols (MCT) in a 1:3 (*w*/*w*) proportion as reported elsewhere [6,10,13]. The MCT oil used mainly comprised caprylic acid (C8), which is a saturated fatty acid. The dilution of SSO in such a saturated oil considerably increases the oxidative stability due to the resulting reduction in the degree of unsaturation. The antioxidant concentration assayed was 0.06 mmol kg^−1^, which is an intermediate concentration compared to those used by other authors [6,13,14]. For comparative purposes, Trolox was also tested at the same experimental conditions. The AE was evaluated by the Rancimat test at 100 °C and by the oven test, this time at 60 °C to shorten the time of the experiments. Hydroperoxides were measured to determine the oxidation extent. In addition, the levels of polymers were also analyzed because substantial polymerization is a marker of the onset of advanced or uninhibited oxidation, i.e., when the antioxidants are exhausted [27,28], in a similar way to the measure upon which is based the Rancimat test. Results showed that, similar to Trolox, the antioxidant effectiveness of α-tocopherol also decreased significantly in the presence of PC in a concentration-dependent way, but this time α-tocopherol was more effective in the presence of PC than Trolox (Figure 4). The induction periods prior to the polymerization were coherent with the OSI values by the Rancimat test. Both tests provided the same relative order of stabilities, i.e., Tro-0 > Toc-0 > Toc-50 > Tro-50 > Toc-1000 > Tro-1000, where Tro and Toc represent Trolox and α-tocopherol, respectively, and the number indicates the PC concentration in mg kg^−1^.

The CMC of PC in the blend SSO-MCT was determined in the presence of 0.06 mmol kg^−1^ α-tocopherol. The result obtained was 80 mg kg^−1^ at 60 °C and 100 °C. Thus, it was similar to that found in SSO containing 1.16 mmol kg^−1^ Trolox. A sharp increase in absorbance was observed this time in the presence of tocopherol. Therefore, similar to Trolox, the protecting effect of α-tocopherol was also drastically reduced even at a PC concentration below the CMC.

To assess how the relative concentrations of the antioxidants and PC affected the AE, the molar concentration of PC was estimated using the molecular weight of the dioleylphosphatidylcholine (786.1). In the range of PC solubility (up to 1000 mg kg^−1^), the molar concentration ratios between the antioxidants and PC in Figure 1 and Figure 2 were 18, 3, and 1, respectively. Thus, the concentration of the antioxidants was nearly 20 times higher than that of the PC when the phospholipid was tested at 50 mg kg^−1^. When the concentration of α-tocopherol was ten times reduced in Figure 3, the concentration of the PC was approximately 2 and 10 times higher than that of the antioxidant when it was assayed at 200 and 1000 mg kg^−1^, respectively. At these conditions, the PC only had a marginal effect. However, a drastic decrease in the AE of α-tocopherol was found in the blend SSO-MCT when it was assayed at a lower concentration and even when this was approximately the same as that of the PC (50 mg kg^−1^) (Figure 4). This effect was not observed in SSO when the molar concentration of α-tocopherol was similar to that of PC (1000 mg kg^−1^) in Figure 1. Similarly, when the molar concentration of PC was ca. 10 times higher compared to α-tocopherol in SSO in Figure 3 (50T-1000 or 250T-5000), the decrease in the OSI was minimal. Consequently, these results suggested that the lipid medium also played a role.

### 3.3. Effect of PC on the Antioxidant Effectiveness of α-Tocopherol in Different Lipid Substrates

**Figure 5:**
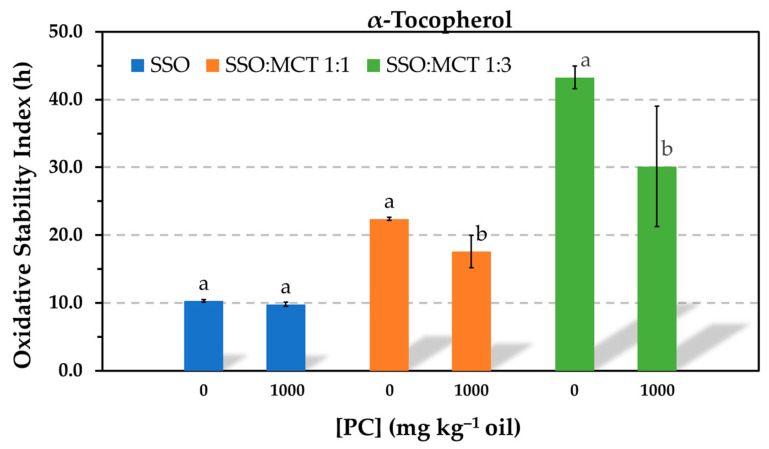
Influence of the addition of 1000 mg kg^−1^phosphatidylcholine (PC) on the antioxidant efficacy of 1.16 mmol kg^−1^ α-tocopherol in stripped sunflower oil (SSO), and 1:1 (*w*/*w*) and 1:3 (*w*/*w*) blends of SSO with MCT, as determined by the Rancimat test at 100 °C. Data represent means (*n* = 3) and standard deviations. For a given lipid medium, different letters indicate significant differences according to Student’s *t*-test (*p* < 0.05).

The effect of PC on the antioxidant effectiveness of 500 mg kg^−1^ α-tocopherol was studied in samples containing different proportions of SSO and MCT. The phospholipid was tested at 1000 mg kg^−1^ to ensure the presence of reverse micelles. The relative molar concentrations of PC and α-tocopherol was ca. 1:1. Results obtained by the Rancimat test showed that when the SSO was diluted with MCT, the PC caused a significant decrease in the AE of α-tocopherol and this was more pronounced in the sample containing more MCT (Figure 5).

**Figure 6:**
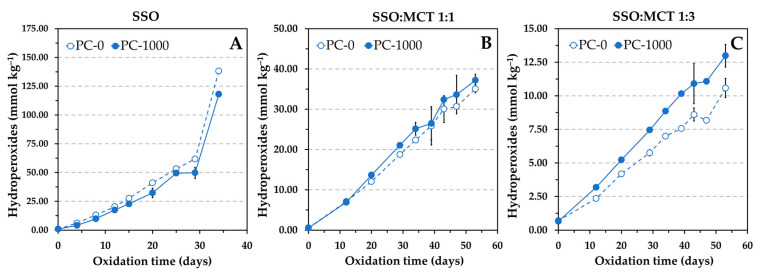
Influence of the addition of 1000 mg kg^−1^ phosphatidylcholine (PC) on the formation of hydroperoxides at 50 °C in different lipid substrates containing 1.16 mmol kg^−1^ α-tocopherol, namely, stripped sunflower oil (SSO) (**A**), and 1:1 (*w*/*w*) (**B**) and 1:3 (*w*/*w*) (**C**) blends of SSO with MCT. Data represent means and standard deviations (*n* = 3). Some of the error bars are within data points.

Similar results were also observed at 50 °C in the oven test, but the effect of PC, as determined by hydroperoxides measurements, was not as pronounced as the Rancimat test showed. Results for hydroperoxides only exhibited clear differences between the control sample and that containing the PC in the lipid mixture containing more MCT (Figure 6). Oxidation occurred more rapidly in the presence of PC, indicating a reduction in the AE of α-tocopherol when the SSO was diluted with MCT.

### 3.4. Effect of PC on the Antioxidant Efficacy of Gallic Acid, Propyl Gallate, and Dodecyl Gallate in SSO

Gallic acid (GA) is a natural phenolic antioxidant found in a number of fruits and in teas [29]. Its molecular structure includes three hydroxy groups at the 3, 4, and 5 positions of the aromatic ring and, similar to Trolox, a carboxylic group. These structural elements contribute to its high polarity and water solubility [30]. GA was also tested in the present study to investigate the significant reductions in AE observed for Trolox in the presence of PC. Specifically, the examination aimed to determine if the carboxylic group played a role in the pronounced effect of PC on Trolox, and GA was employed as a model for this evaluation. For comparative purposes, propyl gallate (PG) and dodecyl gallate (DG) were also examined. In both of these antioxidants, the carboxylic group is esterified and, consequently, blocked with alkyl alcohols, which reduces the polarity of GA. In the case of DG, the alcohol also imparts amphiphilic properties to it. All three antioxidants were evaluated in the Rancimat test at an equivalent molar concentration of 0.59 mmol kg^−1^. Considering the same CMC found for the PC in SSO containing Trolox (60–80 mg kg^−1^), the impact of PC on the AE of GA and its esters was assessed using PC concentrations below (50 mg kg^−1^) and above (1000 mg kg^−1^) the CMC.

**Figure 7:**
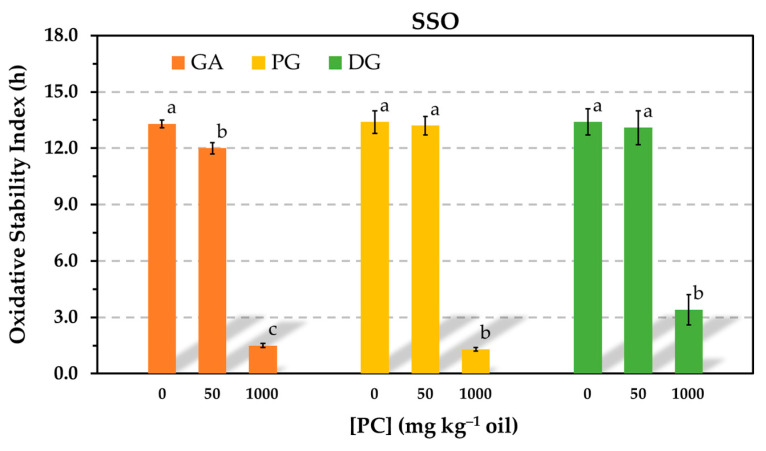
Influence of the phosphatidylcholine (PC) concentration on the oxidative stability index determined in the Rancimat test at 100 °C in stripped sunflower oil containing 0.59 mmol kg^−1^ gallic acid (GA), propyl gallate (PG) or dodecyl gallate (DG). Data represent means and standard deviations (*n* = 3). Different letters for a given antioxidant indicate significant differences according to Duncan’s test (*p* < 0.05).

Significant reductions in AE were observed for all three antioxidants when the PC concentration exceeded the CMC. However, when the concentration of PC was below the CMC, significant differences were only observed for GA. The OSI also decreased by the addition of 50 mg kg^−1^ PC, showing a similar pattern to that found for Trolox in Figure 1. In contrast, the OSI of the samples containing the ester derivatives of GA was unaffected by the addition of 50 mg kg^−1^ of PC (Figure 7).

### 3.5. Influence of PC Concentration on the Oxidative Stability of Neat Sunflower Oil (Non-Purified)

**Figure 8:**
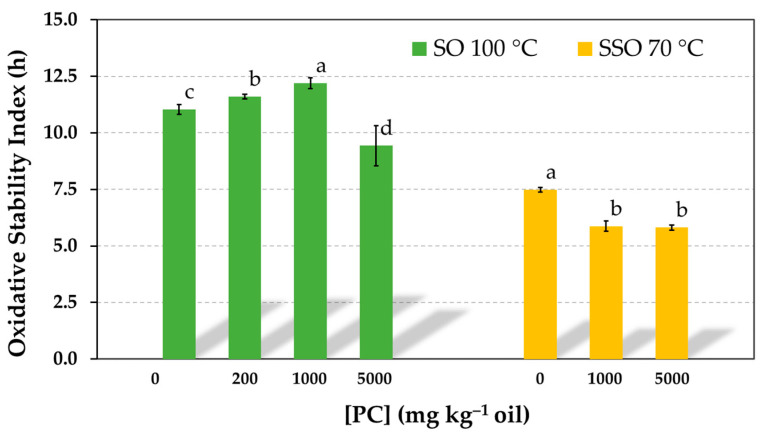
Influence of the phosphatidylcholine (PC) concentration on the oxidative stability index of refined sunflower oil (SO) and stripped sunflower oil in the absence of antioxidants determined in the Rancimat test at 100 °C and 70 °C, respectively. Data represent means and standard deviations (*n* = 3). Different letters indicate significant differences according to Duncan’s test (*p* < 0.05).

To contrast the fact that the addition of PC over a wide range of concentrations had no impact on the AE of α-tocopherol when this was tested at a concentration normally found in sunflower oils (Figure 1 and Figure 2), the effect of PC on the oxidative stability of neat or non-purified sunflower oil was investigated by the Rancimat test. Results showed a small effect on OSI in the concentration range in which the PC was soluble in the oil (up to 1000 mg kg^−1^). A very slight but significant increase was observed (Figure 8). In contrast, the OSI was reduced when the PC concentration was as high as 5000 mg kg^−1^. Similarly, when the effect of PC was studied on SSO in the absence of antioxidants, the OSI also decreased moderately, showing a prooxidant role of PC reverse micelles, as reported in various studies [3,5,6].

## 4. Discussion

The prooxidative role of reverse micelles in bulk oils has been demonstrated in numerous studies (e.g., [3,5,6]). It has been suggested that association colloids increase the prooxidative activity of transition metals [7]. Phospholipids can concentrate at the oil–water interface and attract metals owing to their capability of producing negatively charged interfacial regions [31]. These interfacial metals can interact with lipid hydroperoxides that can migrate and locate at the interface owing to their interfacial activity, catalyzing the hydroperoxide decomposition into free radicals [11]. Therefore, reverse micelles appear to facilitate the interactions between metals and hydroperoxides.

The results obtained in this study for α-tocopherol, as shown in Figure 1 and Figure 2, where the AE remained unchanged in the presence of PC, suggest that the antioxidant somehow prevented the interactions between hydroperoxides and metals. In addition, these results also indicate that the polyunsaturated fatty acyl chains of the PC did not have a significant effect on the oxidation rate of the SSO, probably due to the relatively low PC concentrations.

When Trolox was tested, losses of AE were observed in the presence of PC, but their efficacy was still higher compared to α-tocopherol. To explain the different effects observed for the PC on the effectiveness of these antioxidants, we postulate the hypothesis that main oxidation reactions did not necessarily occur at pre-existing PC reverse micelles, but at reverse micelles mainly formed by hydroperoxides and other oxidation compounds. Lipid hydroperoxides could mainly form in the continuous lipid phase, in a similar way to when the PC is not present in a purified oil. Hydroperoxides are also amphiphilic molecules with interfacial activity that can associate with other interfacial components like the PC to form mixed reverse micelles and/or associate themselves to form pure micelles, as it has been suggested by other authors [3,14,32,33]. Budilarto and Kamal-Eldin [34] demonstrated the formation of colloids during the course of oxidation of purified sunflower or canola oil, i.e., in the absence of pre-existing reverse micelles. They found that the water content and the size of reverse micelles formed by hydroperoxides and other oxidation compounds increased during the induction period, defined as the period prior to an exponential increase of hydroperoxides. The increase in the water content was attributed to the bimolecular decomposition of hydroperoxides.

The capability of hydroperoxides and other lipid oxidation products to self-aggregate in the presence of pre-existing PC reversed micelles could be justified by their less surface activity [3,11]. It is thus evident that such interactions are going to depend on the hydroperoxide concentrations and that they would be more favored as oxidation proceeds. The greater affinity of polar antioxidants for these new interfacial colloids, where lipid oxidation compounds are concentrated and hypothetically lipid radicals are mainly formed, would explain their greater antioxidant effectiveness compared to lipophilic antioxidants. This was definitely the case of Trolox compared to α-tocopherol when these antioxidants were assayed in the absence of PC in the present study and in other works [6,13].

When the PC was added, the results of this study point out that the more polar antioxidant was less available to inhibit lipid oxidation, probably due to a greater affinity for PC compared to the less polar antioxidant. In this regard, Chen et al. [6] demonstrated by fluorescence measurements the incorporation of Trolox into DOPC reverse micelles. It was stated that Trolox partitioned into the water core of the micelles due to its water solubility. The partitioning of Trolox between micelles of PC and micelles of triacylglycerol-hydroperoxides appears to account for the loss of its AE.

Regarding α-tocopherol, its lower affinity for the PC micelles seemed not to affect its location to a great extent and so its availability to inhibit lipid oxidation. The low affinity of α-tocopherol for DOPC reverse micelles has been demonstrated in different studies. Compared to Trolox, Chen et al. [6] found a lower interaction of α-tocopherol with a fluorescence probe, namely, *N*-(7-nitrobenz-2-oxa-1,3-diazol-4-yl)-1,2-dihexadecanoyl-*sn*-glycero-3-phosphoethanolamine (NBD-PE), embedded into DOPC micelles. They stated that α-tocopherol has virtually no water solubility and, unlike Trolox, it cannot partition into the water phase. At best, it would align at the oil–water interface. Similarly, Rokosik et al. [14] found no interactions of α-tocopherol with DOPC reverse micelles by fluorescence measurements. The fluorescence intensity of α-tocopherol did not change in the presence of DOPC. In addition, the fluorescence intensity of the probe NBD-PE was not affected by the presence of α-tocopherol in a concentration range of 1–500 µmol kg^−1^.

The losses in AE found for α-tocopherol in the presence of PC when the antioxidant concentration was reduced in Figure 4 could have been due to its low and ineffective concentration in the continuous lipid phase as a result of its association with PC. At the antioxidant concentration assayed (0.06 mmol kg^−1^), the PC concentration had a greater effect on the effectiveness of Trolox (Figure 4), and α-tocopherol was more effective. This fact can be attributed to the greater affinity of Trolox for the PC. It has been suggested that partitioning of α-tocopherol and Trolox into DOPC micelles could allow the antioxidants to reduce the transition metals to their more prooxidative states and increase the oxidation rate, which would be seen as a decrease in the antioxidant effectiveness [6]. However, this effect was not observed when α-tocopherol was tested at high concentration in Figure 1 and Figure 2.

Most of the studies on the effect of reverse micelles on lipid oxidation employ low antioxidant concentrations to shorten the experiments. As a result, the interactions of antioxidants with reverse micelles are usually extremely favored due to the great differences in concentrations between reverse micelle components and antioxidants. Furthermore, MCT oil is sometimes added to a stripped vegetable oil to have a greater volume of sample. In the present study, it was found that this practice also favored the prooxidative effect of reverse micelles (Figure 5 and Figure 6). The reduction in AE of α-tocopherol in Figure 5 and Figure 6 could be related to an increase in polarity of the lipid medium, since the dielectric constant of MCT oil is a bit larger compared to sunflower oil [35]. The differences in polarity with the lipid medium could determine the migration and location of antioxidants and lipid oxidation compounds and affect their affinities for reverse micelles.

The PC also had an impact on the AE when its concentration was below the CMC. In this regard, losses in AE were observed for Trolox in Figure 1 or for α-tocopherol and Trolox in Figure 4. These results are consistent with the reductions in AE reported for both α-tocopherol and Trolox in SSbO:MCT (1:3, *w*/*w*) due to the addition of 1,2-dibutyryl-*sn*-glycero-3-phosphocholine (DC_4_PC), a synthetic phospholipid that cannot form reverse micelles [6]. Furthermore, it was hypothesized in this study that Trolox could form a complex with PC through the carboxylic group, similar to what has been described for free fatty acids [36]. Results obtained for GA and its esters when the PC was added at a concentration below the CMC, considered to be 60–80 mg kg^−1^, pointed out a role of the carboxylic group (Figure 7). While the AE of GA was reduced in the presence of 50 mg kg^−1^ of PC, no effect was observed for its ester derivatives. As for α-tocopherol, the reduction in its AE caused by the addition of 50 mg kg^−1^ PC in Figure 4 remains unclear. It should be noted that the PC concentration assayed (50 mg kg^−1^) was not much lower than the CMC (60–80 mg kg^−1^), as well as that the CMC was determined by the TCNQ method, which is based on an imprecise determination subjected to the number and selection of data points. Therefore, further research is needed to clarify this matter.

The reductions in AE observed for GA and its alkyl esters (Figure 7) suggested that the polarity of the antioxidant to partition into PC reverse micelles appears to be the predominant factor involved. When the carboxylic group was blocked, the polarity provided by the three hydroxyl groups was still elevated so that strong associations with the PC micelles occurred.

The addition of PC to a commercial sunflower oil had a minor effect on the oil oxidative stability (Figure 8). The prooxidative effect of PC was only observed when its concentration was 5000 mg kg^−1^, significantly exceeding its solubility limit in the oil (1000 mg kg^−1^). Therefore, this fact confirms the low impact of PC reverse micelles on the AE of the α-tocopherol naturally present in sunflower oil.

## 5. Conclusions

At concentrations occurring in refined sunflower seed oils, the AE of α-tocopherol remained unaffected by the presence of PC reverse micelles in SSO. In contrast, the AE of Trolox substantially decreased with the addition of PC in a concentration-dependent way under the same experimental conditions. The location of α-tocopherol appeared not to be significantly affected by the presence of PC reverse micelles, and its availability to inhibit lipid oxidation did not change. However, Trolox seemed to be less available to inhibit lipid oxidation, probably due to its partitioning into PC reverse micelles, as observed in previous studies [6]. The reduction in the AE of Trolox was still greater than that of α-tocopherol when the PC was assayed in a range of concentrations up to 1000 mg kg^−1^, which was its solubility limit in the oil. These results point out that new water–oil interfaces were created by hydroperoxides and other oxidation compounds. Even though Trolox was partitioned into PC reverse micelles losing effectiveness, its greater affinity for micelles consisting of hydroperoxides in comparison with the lipophilic antioxidant could account for its higher effectiveness.

A significant reduction in the AE of α-tocopherol was observed in a blend of SSO and MCT (1:3, *w*/*w*) when the antioxidant concentration was very low, comparable to the concentrations used in most studies in this field [6,13,14], and the molar concentration of PC was similar or 20 times higher than that of the antioxidant. Similarly, Trolox also showed the same pattern, but this time α-tocopherol was more effective in the presence of PC, which can be attributed to its lesser affinity for the PC and so higher effective concentration in the continuous phase to inhibit lipid oxidation. In addition, the prooxidative effect of PC in the mixture of oils, SSO and MCT, was larger compared to SSO. The slightly higher polarity of MCT could have affected the migration and location of antioxidants, and/or lipid oxidation products, as well as their associations with reverse micelles.

It can be concluded that the prooxidative effects of PC observed in different studies that employed model systems consisting of blends of stripped vegetable oils with MCT, along with antioxidants assayed at significantly lower concentrations than PC, are overstated. The effect of PC on the stability of a commercial vegetable oil, such as sunflower oil, which naturally contains substantial amounts of tocopherol, was found to be moderate.

## Data Availability

Data are contained within the article or Supplementary Materials.

## Supplementary Materials

The following supporting information can be downloaded at: https://www.mdpi.com/article/10.3390/antiox12111993/s1, Figure S1: Determination of the critical micelle concentration (CMC) of phosphatidylcholine (PC) in stripped sunflower oil (SSO) in the presence of 1.16 mmol kg^−1^ α-tocopherol (Toc) or Trolox at 100 °C. Toc 1 and Toc 2 refer to duplicate experiments made in different days.
