# Supplementary material for: New Insights into the Loss of Antioxidant Effectiveness of Phenolic Compounds in Vegetable Oils in the Presence of Phosphatidylcholine
Source: Antioxidants (Basel). 2023 Nov 11;12(11):1993. doi: 10.3390/antiox12111993 (PMC10669491; doi:10.3390/antiox12111993)
Supplement: Supplementary file 1 [file antioxidants-12-01993-s001.zip › antioxidants-2670397-supplementary.pdf]

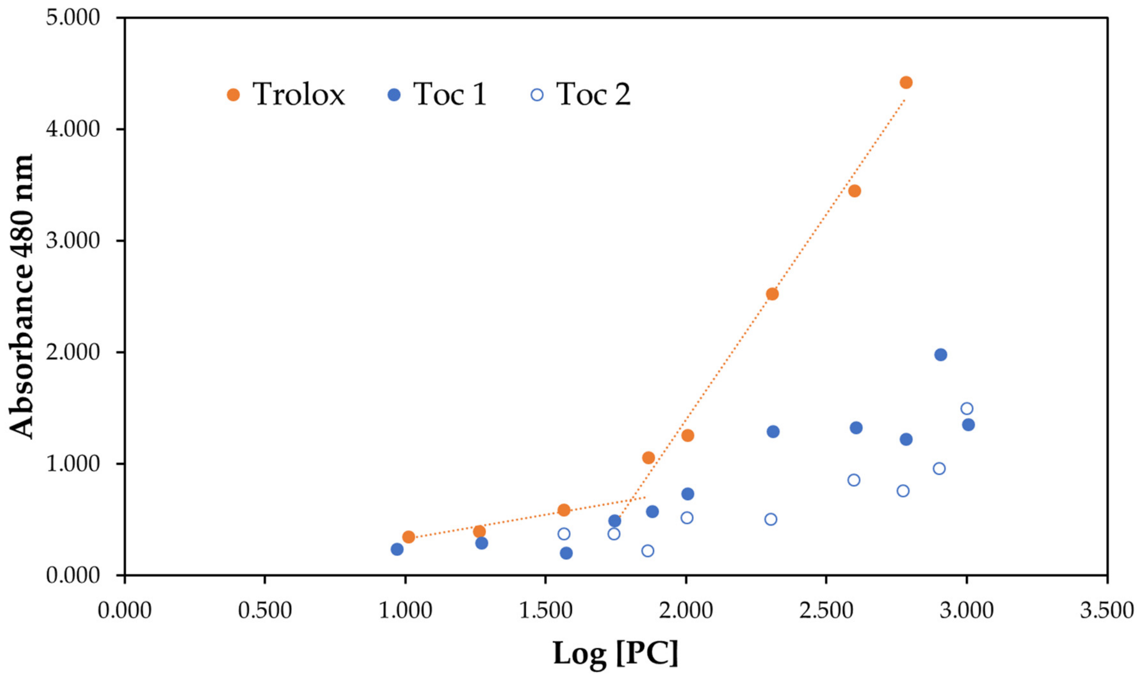

Figure S1. Determination of the critical micelle concentration (CMC) of phosphatidylcholine (PC) in stripped sunflower oil (SSO) in the presence of 1.16 mmol kg^{-1} α-tocopherol (Toc) or Trolox at 100°C. Toc 1 and Toc 2 refer to duplicate experiments made in different days.
